# Supplementary material for: EEG microstates are a candidate endophenotype for schizophrenia
Source: Nat Commun. 2020 Jun 18;11:3089. doi: 10.1038/s41467-020-16914-1 (PMC7303216; doi:10.1038/s41467-020-16914-1)
Supplement: Supplementary file 1 — Supplementary Information [file 41467_2020_16914_MOESM1_ESM.pdf]

Supplementary Information for:

EEG microstates are a candidate endophenotype for schizophrenia

da Cruz et al.

Supplementary Tables

Supplementary Table 1 – Spatial correlation coefficients between microstate class topographies in each pair of groups.

Microstate class	Patients vs Siblings	Patients vs Controls	Controls vs Siblings
A	0.98	0.98	0.97
B	0.97	0.99	0.97
C	0.99	0.98	0.99
D	0.94	0.92	0.97

The high coefficients indicate that the microstate classes were similar between groups.

Supplementary Table 2 – Topographical ANOVA results between microstate class topographies in each pair of groups (*p*-values are not corrected for multiple comparisons).

Microstate class	Patients vs Siblings	Patients vs Controls	Controls vs Siblings
A	0.591	0.500	0.247
B	0.047	0.509	0.110
C	0.145	0.374	0.240
D	0.351	0.280	0.502

We performed topographical ANOVAs (TANOVAs)[1,2] to investigate whether there were systematic group differences in the microstates classes. The only statistical significant group difference was found between patients and siblings for microstate class B (p=0.047). However, this difference disappears after correcting for multiple comparisons using Bonferroni-Holm (p=0.188).

Supplementary Table 3 – Group average statistics (±SD) of the Patients with Schizophrenia, their Unaffected Siblings and Healthy Controls as well as the average difference score of Patients_32 and their paired Siblings_32 for all the computed microstates parameters and classes.

Microstate Parameter	Microstate Class	Group			Patients_32 - Siblings_32 (Δ)
		Patients	Siblings	Controls	
Mean Duration (ms)	A	69.05 ± 10.06	66.93 ± 8.61	71.83 ± 15.20	2.45 ± 11.45
	B	66.34 ± 8.97	74.81 ± 11.87	71.05 ± 8.52	-7.21 ± 12.50
	C	106.99 ± 27.75	99.05 ± 22.16	90.31 ± 18.05	-0.93 ± 25.40
	D	70.42 ± 11.16	71.41 ± 11.02	82.01 ± 16.84	-3.17 ± 15.96
Time Coverage (%)	A	18.21 ± 8.98	15.75 ± 6.79	18.85 ± 11.03	4.32 ± 9.94
	B	16.15 ± 7.71	22.48 ± 9.99	19.27 ± 7.35	-3.69 ± 11.06
	C	46.38 ± 12.98	41.68 ± 12.70	34.00 ± 11.87	0.82 ± 16.71
	D	19.27 ± 8.38	20.10 ± 7.73	27.89 ± 11.61	-1.45 ± 11.79
Occurrence	A	1.87 ± 0.66	1.69 ± 0.62	1.82 ± 0.61	0.38 ± 0.81
	B	1.74 ± 0.58	2.12 ± 0.59	1.93 ± 0.53	-0.18 ± 0.79
	C	2.83 ± 0.38	2.74 ± 0.36	2.52 ± 0.45	0.04 ± 0.43
	D	1.95 ± 0.59	2.00 ± 0.58	2.34 ± 0.59	-0.04 ± 0.80

Supplementary Table 4 - Two-sided Pearson correlation results of the Patients_32 - Siblings_32 pairs for all the computed microstates parameters and classes. Statistically significant results (without Bonferroni-Holm correction) are indicated in bold. No statistically significant results were found after correction for multiple comparisons.

Microstate Parameter	Microstate Class	*r*(30) - value	*p* - value	p_{holm} - value
Mean Duration	A	0.016	0.930	1.000
	B	**0.360**	**0.043**	0.473
	C	0.043	0.817	1.000
	D	0.090	0.623	1.000
Time Coverage	A	0.032	0.861	1.000
	B	0.314	0.080	0.800
	C	0.109	0.552	1.000
	D	-0.023	0.898	1.000
Occurrence	A	0.056	0.759	1.000
	B	0.120	0.514	1.000
	C	**0.430**	**0.014**	0.168
	D	0.044	0.811	1.000

Supplementary Table 5 - Post-hoc group comparisons (all patients vs. all siblings) of all microstate parameters (mean duration, time coverage, and occurrence), Bonferroni-Holm corrected, for each microstate class (A, B, C, D). Statistically significant differences in bold.

Parameter	Microstate	Patients (n=101) vs. Siblings (n=43)
Mean Duration (ms)	Class A	p=0.863, p_{holm}=1.000, d=0.029, 95% CI [-0.328, 0.386]
	Class B	**p=2.102e-4, p_{holm}=0.003, d=-0.637, 95% CI [-1.000, -0.271]**
	Class C	p=0.058, p_{holm}=0.464, d=0.320, 95% CI [-0.039, 0.678]
	Class D	p=0.147, p_{holm}=0.959, d=-0.247, 95% CI [-0.604, 0.111]
Time Coverage (%)	Class A	p=0.435, p_{holm}=1.000, d=0.127, 95% CI [-0.230, 0.484]
	Class B	**p=0.002, p_{holm}=0.022, d=-0.523, 95% CI [-0.884, -0.160]**
	Class C	p=0.017, p_{holm}=0.153, d=0.408, 95% CI [0.047, 0.767]
	Class D	p=0.154, p_{holm}=0.959, d=-0.238, 95% CI [-0.596, 0.120]
Occurrence	Class A	p=0.587, p_{holm}=1.000, d=0.090, 95% CI [-0.230, 0.484]
	Class B	p=0.006, p_{holm}=0.060, d=-0.473, 95% CI [-0.833, -0.111]
	Class C	p=0.240, p_{holm}=0.960, d=0.201, 95% CI [-0.157, 0.558]
	Class D	p=0.137, p_{holm}=0.959, d=-0.247, 95% CI [-0.605, 0.111]

Since groups (all patients and all siblings) differed in age (t(142)=3.007, p=0.003) and gender (χ^2(1)=25.126, p=5.371e-7) but not in education (t(142)=1.430, p=0.155) or handedness (χ^2(1)=0.096, p=0.757), gender was used as a factor and age as a covariate in subsequent analyses. For each of microstates parameter, we performed a three-way repeated measures ANOVA, with three factors: gender, group, and microstate class. The analyses showed non-significant Gender × Group × Microstate Class interaction for mean duration (F(3,417)=1.066, P=0.363, η^2=0.006, 90% CI [<0.001, 0.021]), time of coverage (F(3,417)=1.077, P=0.358, η^2=0.007, 90% CI [<0.001, 0.021]), and occurrence (F(3,417)=0.770, P=0.511, η^2=0.004, 90% CI [<0.001, 0.016]). The analyses also yielded significant Microstate Class × Group interaction for mean duration (F(3,417)=4.845, P=0.003, η^2=0.027, 90% CI [0.007, 0.061]), time of coverage (F(3,417)=4.883, P=0.002, η^2=0.030, 90% CI [0.007, 0.062]), and occurrence (F(3,417)=3.449, P=0.017, η^2=0.016, 90% CI [0.002, 0.048]). Post-hoc pairwise group comparisons, using Bonferroni-Holm correction for the 12 group comparisons, showed that siblings had increased mean duration and time coverage of microstate class B compared to patients. P values refer to main effects of Group (F(1,139)) following Group × Gender ANCOVAs with age as a covariate. η^2's were converted to Cohen's d's. Since, after Bonferroni-Holm correction, occurrence of microstate class B in siblings was marginally significantly higher than in patients, we conducted a JZS Bayes ANCOVA with default priors[3–5] to evaluate if there was more evidence in favor of the null hypothesis (no difference between groups) as compared to the alternative (i.e., that there was a difference between groups). Results indicated that the model with the Group effect was preferred to the models with Group + Gender, Group + Gender + Group × Gender, Group + Gender + Age, Group + Gender + Age + Group × Gender, and the Null model by Bayes factors of 4.255, 12.821, 20.408, 35.714, and 50, respectively.

Supplementary Table 6 - Group average statistics (±SD) of the patients with schizophrenia without siblings in the current study (Patients_no_Sibs) for all the computed microstates parameters (mean duration, time coverage, and occurrence) and classes (A, B, C, and D). As well as post-hoc group comparisons (Patients_no_Sibs vs. Siblings) for all microstate parameters and each microstate class. Statistically significant differences are indicated in bold.

Microstate Parameter	Microstate Class	Patients_no_Sibs (n = 69)	Patients_no_Sibs vs. Siblings (n=43)
Mean Duration (ms)	A	69.01 ± 10.28	p=0.626, p_{holm}=1.000, d=0.090, 95% CI [-0.291, 0.471]
	B	65.89 ± 8.32	**p=2.331e-4, p_{holm}=0.003, d=0.725, 95% CI [0.331, 1.116]**
	C	111.09 ± 26.43	p=0.029, p_{holm}=0.232, d=0.424, 95% CI [0.038, 0.808]
	D	70.78 ± 11.09	p=0.346, p_{holm}=1.000, d=0.180, 95% CI [-0.202, 0.561]
Time Coverage (%)	A	17.61 ± 9.20	p=0.501, p_{holm}=1.000, d=0.127, 95% CI [-0.254, 0.508]
	B	15.11 ± 6.51	**p=3.014e-4, p_{holm}=0.003, d=0.717, 95% CI [0.323, 1.108]**
	C	48.07 ± 12.46	p=0.011, p_{holm}=0.099, d=0.496, 95% CI [0.109, 0.881]
	D	19.21 ± 8.48	p=0.322, p_{holm}=1.000, d=0.191, 95% CI [-0.191, 0.572]
Occurrence	A	1.79 ± 0.66	p=0.772, p_{holm}=1.000, d=0.056, 95% CI [-0.325, 0.437]
	B	1.66 ± 0.52	**p=7.857e-4, p_{holm}=0.008, d=0.667, 95% CI [0.275, 1.056]**
	C	2.84 ± 0.34	p=0.142, p_{holm}=0.994, d=0.286, 95% CI [-0.097, 0.668]
	D	1.94 ± 0.60	p=0.322, p_{holm}=1.000, d=0.191, 95% CI [-0.191, 0.572]

We compared the microstates dynamics of all patients without siblings in the current study (Patients_no_Sibs; n = 69) against all siblings. Since the groups differed in age ($t(110)$=3.724, p=3.114e-4) and gender ($\chi^2(1)$=21.152, p=4.243e-6) but not in education ($t(110)$=1.451, p=0.150) or handedness ($\chi^2(1)$=0.006, p=0.940), gender was used as a factor and age as a covariate in subsequent analyses. For each of the computed microstates parameters, we performed a three-way repeated measures ANOVA, with three factors: gender, group, and microstate class. The analyses showed non-significant Gender × Group × Microstate Class interaction for mean duration ($F(3,321)$=0.357, P=0.784, η^2=0.003, 90% CI [<0.001, 0.011]), time of coverage ($F(3,321)$=0.320, P=0.811, η^2=0.002, 90% CI [<0.001, 0.010]), and occurrence ($F(3,321)$=0.172, P=0.915, η^2=9.971e-4, 90% CI [<0.001, 0.004]). The analyses also yielded significant Microstate Class × Group interaction for mean duration ($F(3,321)$=5.522, P=0.001, η^2=0.039, 90% CI [0.013, 0.086]), time of coverage ($F(3,321)$=5.702, P=8.174e-4, η^2=0.044, 90% CI [0.014, 0.088]), and occurrence ($F(3,321)$=4.303, P=0.005, η^2=0.025, 90% CI [0.007, 0.072]). Post-hoc pairwise group comparisons, using Bonferroni-Holm correction for the 12 comparisons, showed that siblings had increased mean duration, time coverage, and occurrence of microstate class B compared to Patients_no_Sibs. P-values refer to main effects of Group ($F(1,107)$) following Group × Gender ANCOVAs with age as a covariate. η^2's were converted to Cohen's d's

Supplementary Table 7 - Two-sided Pearson correlation between microstate parameters and Chlorpromazine equivalent (CPZ), Scale for Assessment of Negative (SANS) and Positive (SAPS) Symptoms, and Illness duration, for patients with schizophrenia (df - degrees of freedom). Significant correlations (not corrected for multiple comparisons) are in bold.

Microstate Parameter	Microstate Class	CPZ (df = 86)	SANS (df = 99)	SAPS (df = 99)	Illness Duration (df = 99)
Mean Duration	A	*r*=-0.015, *p*=0.890	*r*=0.018, *p*=0.862	*r*=0.007, *p*=0.948	*r*=0.069, *p*=0.490
	B	*r*=-0.057, *p*=0.596	*r*=0.056, *p*=0.576	*r*=0.041, *p*=0.685	*r*=-0.062, *p*=0.539
	C	*r*=0.012, *p*=0.910	*r*=-0.017, *p*=0.866	*r*=-0.066, *p*=0.514	*r*=-0.053, *p*=0.598
	D	*r*=0.092, *p*=0.391	*r*=-0.144, *p*=0.150	*r*=0.004, *p*=0.972	*r*=0.042, *p*=0.674
Time Coverage	A	*r*=-0.041, *p*=0.702	*r*=0.028, *p*=0.778	*r*=0.026, *p*=0.794	*r*=0.089, *p*=0.374
	B	*r*=-0.098, *p*=0.366	*r*=0.119, *p*=0.237	*r*=0.039, *p*=0.702	*r*=-0.065, *p*=0.516
	C	*r*=0.038, *p*=0.724	*r*=0.032, *p*=0.749	*r*=-0.048, *p*=0.635	*r*=-0.060, *p*=0.552
	D	*r*=0.079, *p*=0.465	*r*=-0.190, *p*=0.057	*r*=0.010, *p*=0.918	*r*=0.057, *p*=0.571
Occurrence	A	*r*=-0.011, *p*=0.921	*r*=0.034, *p*=0.734	*r*=0.045, *p*=0.653	*r*=0.080, *p*=0.426
	B	*r*=-0.062, *p*=0.567	*r*=0.121, *p*=0.228	*r*=0.037, *p*=0.717	*r*=-0.073, *p*=0.469
	C	***r*=0.236, *p*=0.027**	*r*=0.010, *p*=0.919	*r*=0.033, *p*=0.743	*r*=-0.085, *p*=0.397
	D	*r*=0.081, *p*=0.453	*r*=-0.183, *p*=0.067	*r*=0.017, *p*=0.863	*r*=0.036, *p*=0.720

In patients with schizophrenia, CPZ equivalents were found to correlate with the occurrence of microstate class C. However, the correlation was not significant after correction for 12 comparisons (4 microstate classes × 3 parameters) with Bonferroni-Holm ($r(86)=0.236$, $p=0.027$, $p_{holm}=0.324$). No other significant associations were found between the computed microstate parameters and either CPZ equivalent, SANS, SAPS, or illness duration.

Supplementary Table 8 – Spatial correlation coefficients and topographical ANOVA (TANOVA) between microstate class topographies of FEP and Patients_22 (*p*-values are not corrected for multiple comparisons).

Microstate Class	Spatial Correlation Coefficients	TANOVA *p*-values
A	0.96	0.705
B	0.97	0.929
C	0.99	0.800
D	0.91	0.559

The high coefficients indicate that the microstate classes were similar between groups.

We performed TANOVAs[1,2] to investigate whether there were systematic group differences in the microstates classes of FEP and Patients_22. No statistical significant group differences were found.

Supplementary Table 9 – Group average statistics (±SD) of the FEP and Patients_22 groups for all the computed microstates parameters and classes.

Microstate Parameter	Microstate Class	Group	
		FEP	Patients_22
Mean Duration (ms)	A	69.97 ± 18.62	66.74 ± 9.86
	B	78.07 ± 39.85	67.71 ± 10.00
	C	100.80 ± 22.48	103.85 ± 13.08
	D	68.50 ± 11.55	68.90 ± 8.91
Time Coverage (%)	A	17.51 ± 12.58	16.73 ± 7.32
	B	21.34 ± 14.75	17.74 ± 8.25
	C	43.08 ± 16.43	46.91 ± 8.32
	D	18.07 ± 9.10	18.63 ± 8.53
Occurrence	A	1.73 ± 0.61	1.78 ± 0.61
	B	1.92 ± 0.53	1.83 ± 0.54
	C	2.68 ± 0.62	2.94 ± 0.23
	D	1.88 ± 0.59	1.91 ± 0.63

Supplementary Table 10 - Two-sided Pearson correlation between all computed microstate parameters and Chlorpromazine equivalent (CPZ), Scale for Assessment of Negative (SANS) and Positive (SAPS) Symptoms, and Illness duration, for patients with a first episode of psychosis (FEP). (df - degrees of freedom). Statistically significant correlations (without correction for multiple comparisons) are indicated in bold.

Microstate Parameter	Microstate Class	CPZ (df = 18)	SANS (df = 20)	SAPS (df = 20)	Illness Duration (df = 20)
Mean Duration	A	*r*=-0.154, *p*=0.517	*r*=0.197, *p*=0.379	*r*=0.179, *p*=0.426	*r*=0.117, *p*=0.605
	B	*r*=-0.125, *p*=0.600	*r*=-0.054, *p*=0.812	*r*=-0.126, *p*=0.576	*r*=-0.110, *p*=0.627
	C	*r*=0.242, *p*=0.304	*r*=0.106, *p*=0.638	*r*=0.170, *p*=0.449	*r*=-0.193, *p*=0.390
	D	*r*=-0.178, *p*=0.452	*r*=-0.352, *p*=0.109	*r*=0.142, *p*=0.529	*r*=-0.230, *p*=0.303
Time Coverage	A	*r*=-0.156, *p*=0.511	*r*=0.244, *p*=0.273	*r*=0.150, *p*=0.504	*r*=0.205, *p*=0.359
	B	*r*=-0.127, *p*=0.595	*r*=0.055, *p*=0.808	*r*=-0.150, *p*=0.506	*r*=0.033, *p*=0.884
	C	*r*=0.240, *p*=0.309	*r*=0.049, *p*=0.828	*r*=0.069, *p*=0.759	*r*=-0.121, *p*=0.590
	D	*r*=-0.030, *p*=0.901	***r*=-0.516, *p*=0.014**	*r*=-0.091, *p*=0.688	*r*=-0.118, *p*=0.600
Occurrence	A	*r*=-0.103, *p*=0.666	*r*=0.281, *p*=0.205	*r*=0.048, *p*=0.832	*r*=0.212, *p*=0.344
	B	*r*=-0.061, *p*=0.799	*r*=0.390, *p*=0.073	*r*=0.047, *p*=0.834	*r*=0.410, *p*=0.058
	C	*r*=0.134, *p*=0.573	*r*=0.075, *p*=0.741	*r*=0.079, *p*=0.728	*r*=0.084, *p*=0.709
	D	*r*=0.033, *p*=0.890	***r*=-0.429, *p*=0.046**	*r*=-0.149, *p*=0.507	*r*=0.045, *p*=0.844

In FEP, the SANS scores correlated negatively with the time coverage and occurrence of microstate class D. However, the correlations were not significant after correction for 12 comparisons (4 microstate classes × 3 microstate parameters) with Bonferroni-Holm. No other significant associations were found between the computed microstate parameters and either CPZ equivalent, SANS, SAPS, or illness duration.

Supplementary Table 11 - Group average statistics (±SD) of the patients with a first episode of psychosis that participated in the first and second testing sessions (FEP_2), and all the three testing sessions (FEP_3) for all the computed microstate parameters.

Microstate Parameter	Microstate Class	FEP_2 (n=16)		FEP_3 (n=11)		
		Testing Session		Testing Session		
		First	Second	First	Second	Third
Mean Duration (ms)	A	71.63 ± 21.62	67.31 ± 10.61	72.59 ± 25.85	65.40 ± 9.49	66.84 ± 5.00
	B	69.93 ± 5.52	70.34 ± 6.99	69.81 ± 5.20	68.88 ± 6.00	69.03 ± 5.53
	C	99.68 ± 19.38	101.44 ± 17.68	103.61 ± 21.92	104.78 ± 19.15	112.68 ± 43.22
	D	70.09 ± 12.44	67.02 ± 11.59	66.54 ± 7.15	65.43 ± 8.62	65.24 ± 10.87
Time Coverage (%)	A	19.57 ± 14.20	18.07 ± 8.38	19.54 ± 16.61	16.72 ± 6.92	17.09 ± 7.03
	B	18.69 ± 6.06	20.91 ± 5.83	18.90 ± 5.70	20.30 ± 4.62	19.64 ± 7.66
	C	42.45 ± 13.60	44.08 ± 10.40	45.13 ± 15.50	47.37 ± 10.46	47.58 ± 14.62
	D	19.28 ± 9.40	16.95 ± 8.83	16.43 ± 6.82	15.60 ± 6.29	15.69 ± 7.81
Occurrence	A	1.85 ± 0.66	1.90 ± 0.75	1.76 ± 0.63	1.82 ± 0.71	1.85 ± 0.68
	B	1.96 ± 0.54	2.12 ± 0.47	1.97 ± 0.48	2.10 ± 0.41	2.03 ± 0.63
	C	2.74 ± 0.45	2.80 ± 0.35	2.72 ± 0.52	2.84 ± 0.39	2.73 ± 0.49
	D	1.94 ± 0.75	1.77 ± 0.50	1.80 ± 0.53	1.72 ± 0.47	1.70 ± 0.64

Supplementary Table 12 - Subtypes of FEP diagnosis of FEP according to the DSM-IV for all three testing sessions

Testing Session	Number of patients	Diagnosis (DSM-IV)
**First (total n = 22)**		
	2	Schizophrenia, Disorganized Type (295.1)
	16	Schizophrenia, Paranoid Type (295.3)
	3	Schizophrenia, Undifferentiated Type (295.9)
	1	Bipolar I Disorder, Most Recent Episode Depressed, In Partial Remission (296.55)
**Second (total n = 16)**		
	1	Schizophrenia, Disorganized Type (295.1)
	8	Schizophrenia, Paranoid Type (295.3)
	2	Schizoaffective Disorder (295.7)
	3	Schizophrenia, Undifferentiated Type (295.9)
	1	Bipolar I Disorder, Most Recent Episode Depressed, Mild (296.51)
	1	Bipolar I Disorder, Most Recent Episode Depressed, In Partial Remission (296.55)
**Third (total n = 11)**		
	8	Schizophrenia, Paranoid Type (295.3)
	2	Schizophrenia, Undifferentiated Type (295.9)
	1	Bipolar I Disorder, Most Recent Episode Depressed, In Partial Remission (296.55)

Supplementary Table 13 - List of studies identified during the literature search and information whether it was included or excluded from the meta-analysis.

N	Study ID	Excluded	Exclusion Reason	Population
1	Koenig et al., 1999	no		Schizophrenia
2	Lehmann et al., 2005	no		Schizophrenia
3	Kikuchi et al., 2007	no		Schizophrenia
4	Nishida et al., 2013	no		Schizophrenia
5	Andreou et al., 2014	no		FEP
6	Tomescu et al., 2015	yes	same participants as in da Cruz et al., current	Schizophrenia
7	Tomescu et al., 2014	no		22q11
8	Irisawa et al., 2006	yes	3 classes	Schizophrenia
9	Strelets et al., 2003	yes	not 10-20 system, low n of electrodes	Schizophrenia
10	Giordano et al., 2018	no		Schizophrenia
11	Murphy et al., 2019	no		FEP
12	Soni et al., 2019	yes	task-related	Schizophrenia
13	Soni et al., 2018	yes	5 classes	Schizophrenia
14	Sverak et al., 2018	yes	5 classes and TMS	Schizophrenia
15	Rieger et al., 2016	yes	meta-analysis, no original data	
16	Diaz Hernandez et al., 2016	yes	no patients with schizophrenia + neurofeedback	Healthy
17	Khanna et al., 2015	yes	review, no original data	
18	Schlegel et al., 2012	yes	personality traits, skeptical versus believer	Healthy
19	Kindler et al., 2011	yes	no control group, hallucination	Schizophrenia
20	Mucci et al., 2005	yes	schizotypy	Healthy
21	Stevens et al., 1997	yes	task-related and only one microstate	Schizophrenia
22	Kleinlogel et al., 2007	yes	task-related	Schizophrenia
23	Katayama et al., 2007	yes	no patients with schizophrenia, hypnosis	Healthy
24	Yoshimura et al., 2007	yes	heathy participants and drugs	Healthy
25	Kochi et al., 1996	yes	Evoked-related potentials	Schizophrenia
26	Begré et al., 2008	yes	task (working memory)	Schizophrenia
27	Stirk et al., 1995	yes	no patients with schizophrenia	Depressive
28	Michel and Koenig, 2018	yes	review, no original data	
29	da Cruz et al., current	no		Schizophrenia

Supplementary Figures

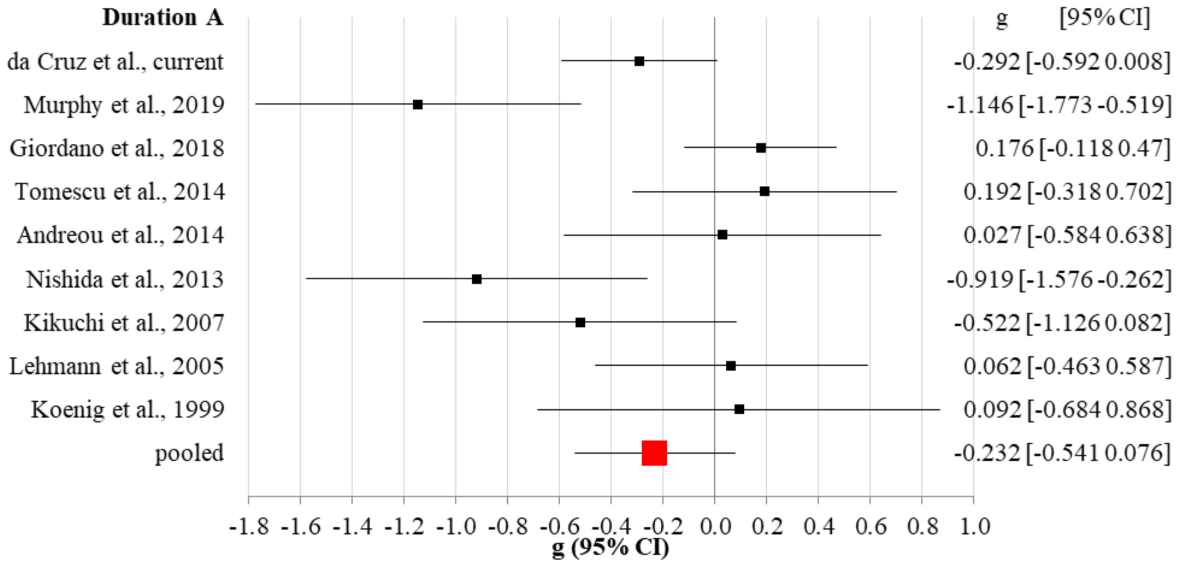

Supplementary Figure 1 - Forest plot of studies considering the mean duration of microstate class A. A two-sided *Z*-test revealed no consistent group differences between patients and controls (N=685, k=9, g=-0.232, 95% CI [-0.541, 0.076], p=0.140, p_{holm}=0.889). *P*-values were corrected for 12 comparisons (3 microstate parameters × 4 microstate classes) using Bonferroni-Holm correction (p_{holm}). I^2 statistics suggests that there is medium variability among studies (i.e., heterogeneity; I^2=68%, p=0.002). Data are presented as Hedge's g and error bars indicate 95% CI. Source data are provided as a Source Data file.

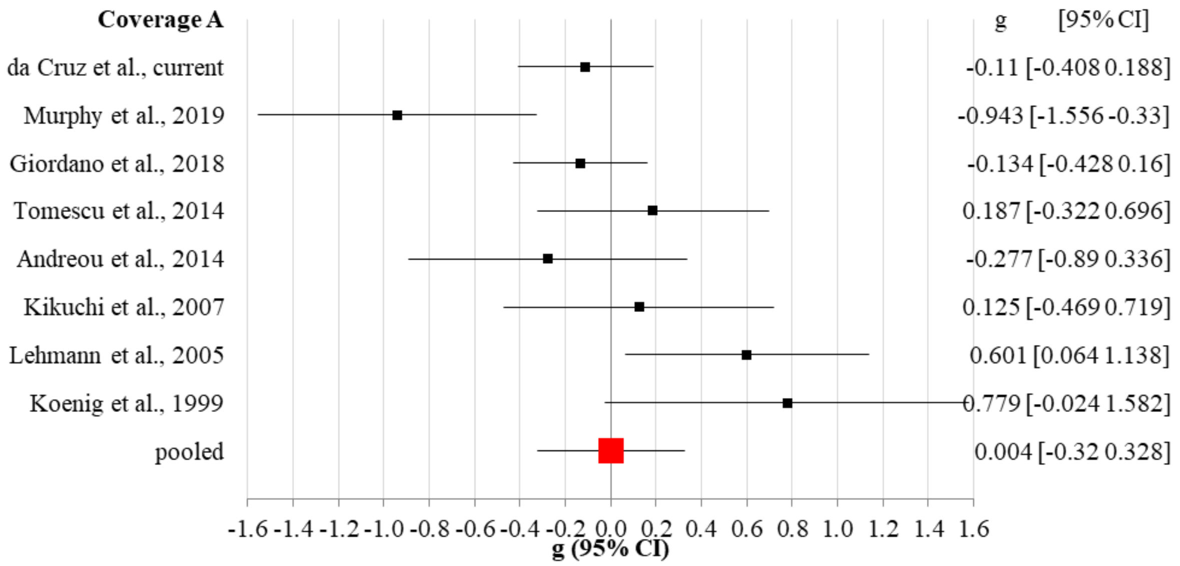

Supplementary Figure 2 - Forest plot of studies considering the time coverage of microstate class A. A two-sided *Z*-test revealed no consistent group differences between patients and controls (N=647, k=8, g=0.004, 95% CI [-0.320, 0.328], p=0.980, p_{holm}=1.000). *P*-values were corrected for 12 comparisons (3 microstate parameters × 4 microstate classes) using Bonferroni-Holm correction (p_{holm}). I^2 statistics suggests that there is medium variability among studies (i.e., heterogeneity; I^2=65%, p=0.006). Data are presented as Hedge's g and error bars indicate 95% CI. Source data are provided as a Source Data file.

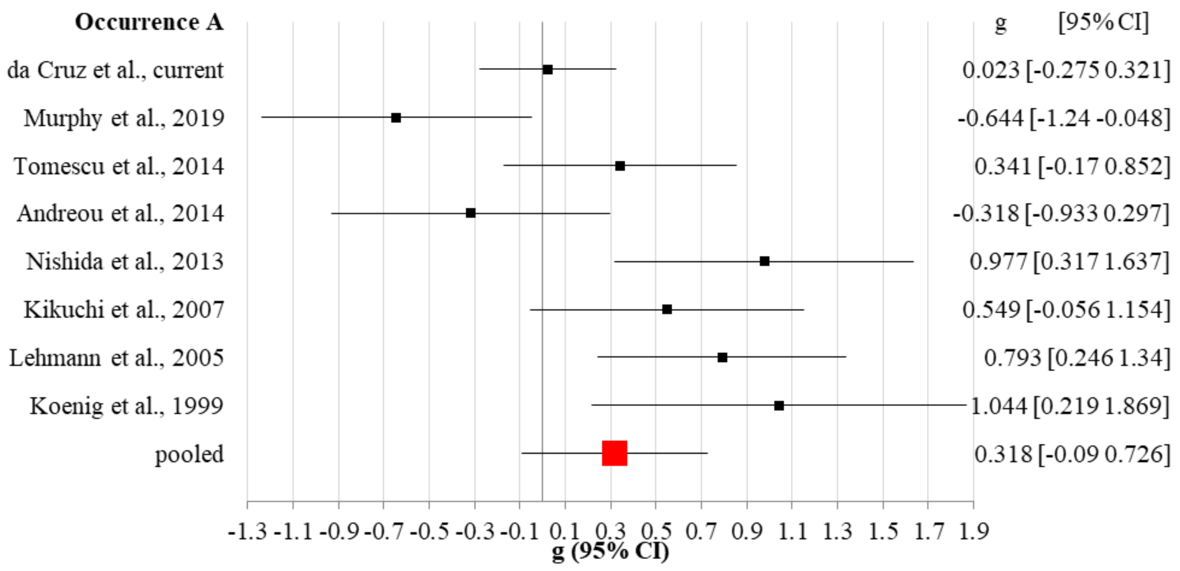

Supplementary Figure 3 - Forest plot of studies considering the occurrence of microstate class A. A two-sided *Z*-test revealed no consistent group differences between patients and controls (N=479, k=8, g=0.318, 95% CI [-0.090, 0.726], p=0.127, p_{holm}=0.889). *P*-values were corrected for 12 comparisons (3 microstate parameters × 4 microstate classes) using Bonferroni-Holm correction (p_{holm}). I^2 statistics suggests that there is medium variability among studies (i.e., heterogeneity; I^2=74%, p=0.0003). Data are presented as Hedge's g and error bars indicate 95% CI. Source data are provided as a Source Data file.

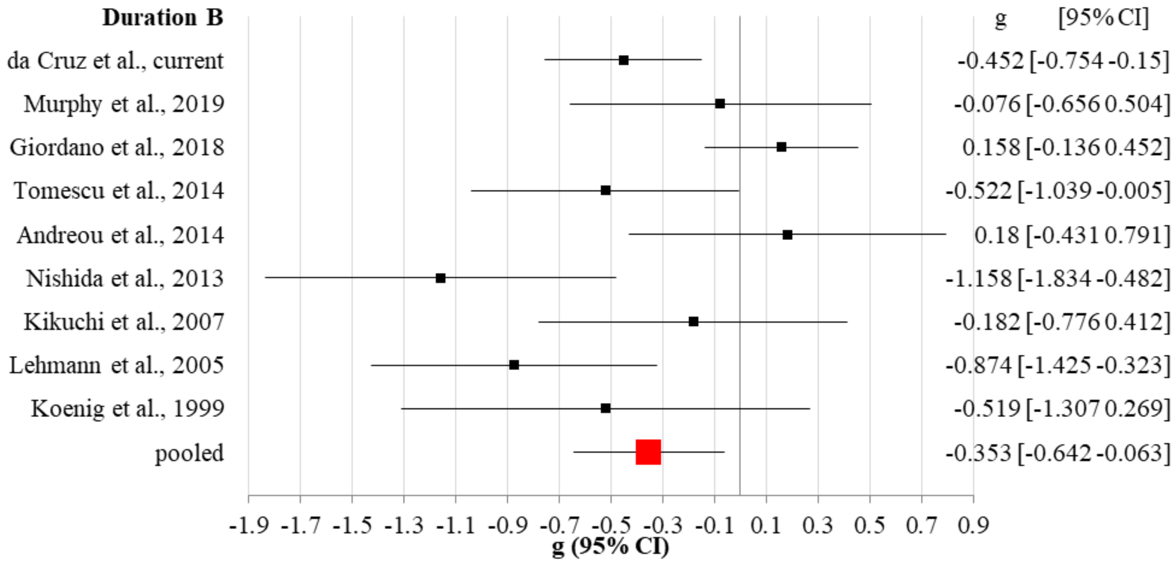

Supplementary Figure 4 - Forest plot of studies considering the mean duration of microstate class B. A two-sided *Z*-test revealed that patients have shorter microstate class B mean durations than controls; however, the result was not significant after correction for multiple comparisons (N=685, k=9, g=-0.353, 95% CI [-0.642, -0.063], p=0.017, p_{holm}=0.136). *P*-values were corrected for 12 comparisons (3 microstate parameters × 4 microstate classes) using Bonferroni-Holm correction (p_{holm}). I^2 statistics suggests that there is medium variability among studies (i.e., heterogeneity; I^2=68%, p=0.002). Data are presented as Hedge's g and error bars indicate 95% CI. Source data are provided as a Source Data file.

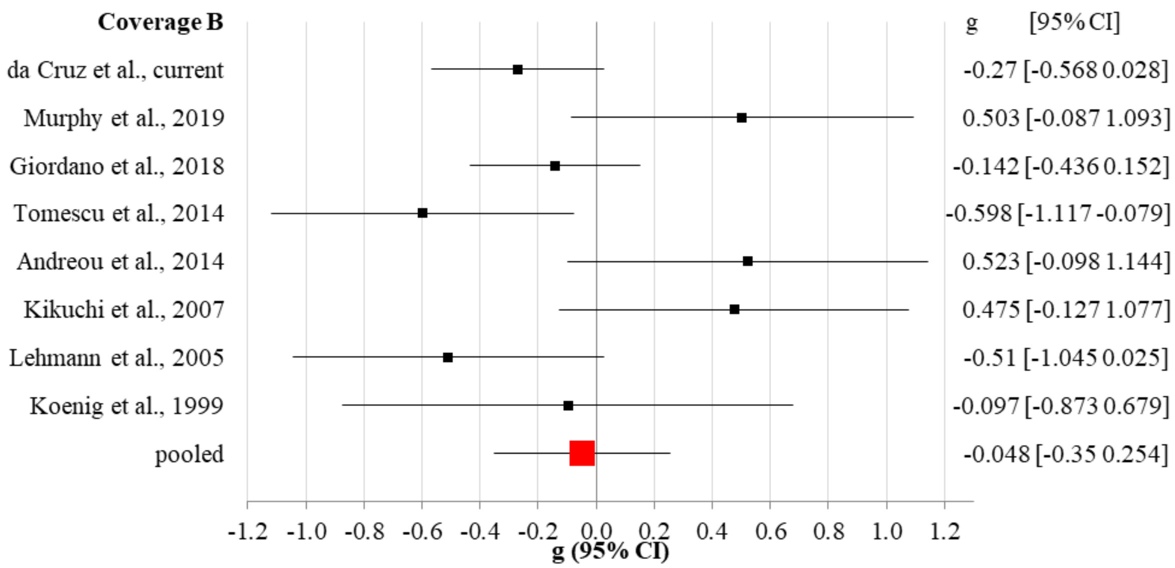

Supplementary Figure 5 - Forest plot of studies considering the time coverage of microstate class B. A two-sided *Z*-test revealed no consistent group differences between patients and controls (N=647, k=8, g=-0.048, 95% CI [-0.350, 0.254], p=0.754, p_{holm}=1.000). *P*-values were corrected for 12 comparisons (3 microstate parameters × 4 microstate classes) using Bonferroni-Holm correction (p_{holm}). I^2 statistics suggests that there is medium variability among studies (i.e., heterogeneity; I^2=62%, p=0.010). Data are presented as Hedge's g and error bars indicate 95% CI. Source data are provided as a Source Data file.

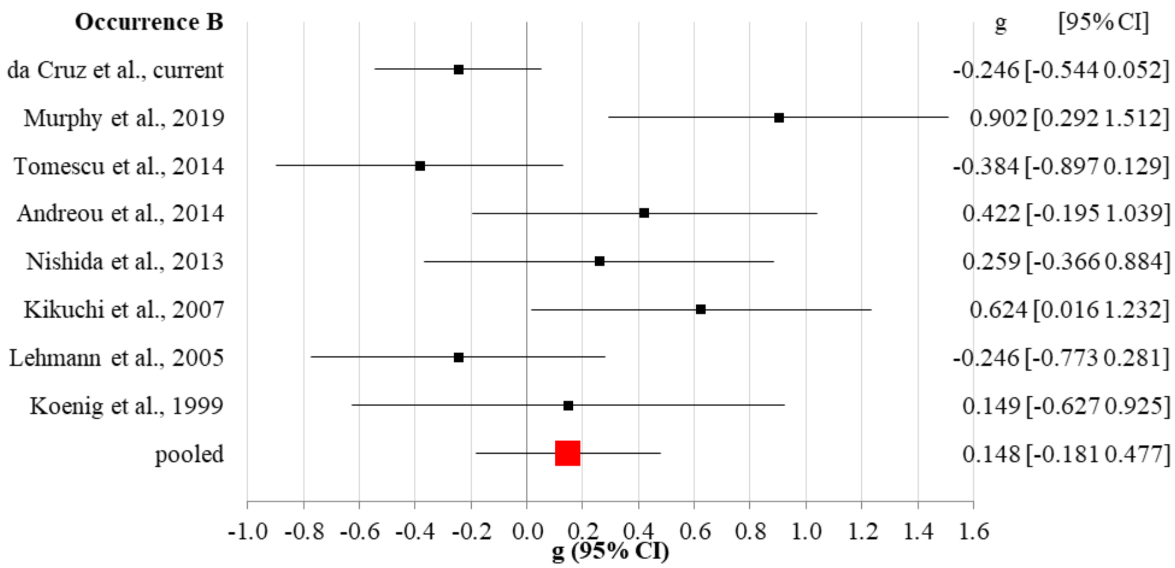

Supplementary Figure 6 - Forest plot of studies considering the occurrence of microstate class B. A two-sided *Z*-test revealed no consistent group differences between patients and controls (N=479, k=8, g=0.148, 95% CI [-0.181, 0.477], p=0.378, p_{holm}=1.000). *P*-values were corrected for 12 comparisons (3 microstate parameters × 4 microstate classes) using Bonferroni-Holm correction (p_{holm}). I^2 statistics suggests that there is medium variability among studies (i.e., heterogeneity; I^2=66%, p=0.005). Data are presented as Hedge's g and error bars indicate 95% CI. Source data are provided as a Source Data file.

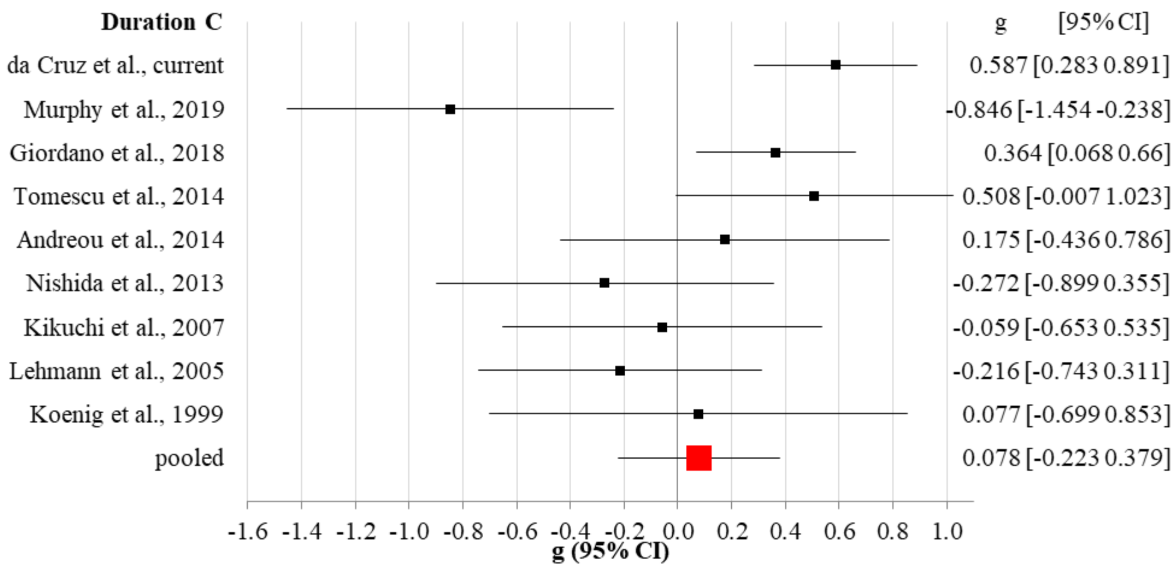

Supplementary Figure 7 - Forest plot of studies considering the duration of microstate class C. A two-sided *Z*-test revealed no consistent group differences between patients and controls (N=685, k=9, g=0.078, 95% CI [-0.223, 0.379], p=0.611, p_{holm}=1.000). *P*-values were corrected for 12 comparisons (3 microstate parameters × 4 microstate classes) using Bonferroni-Holm correction (p_{holm}). I^2 statistics suggests that there is medium variability among studies (i.e., heterogeneity; I^2=69%, p=0.001). Data are presented as Hedge's g and error bars indicate 95% CI. Source data are provided as a Source Data file.

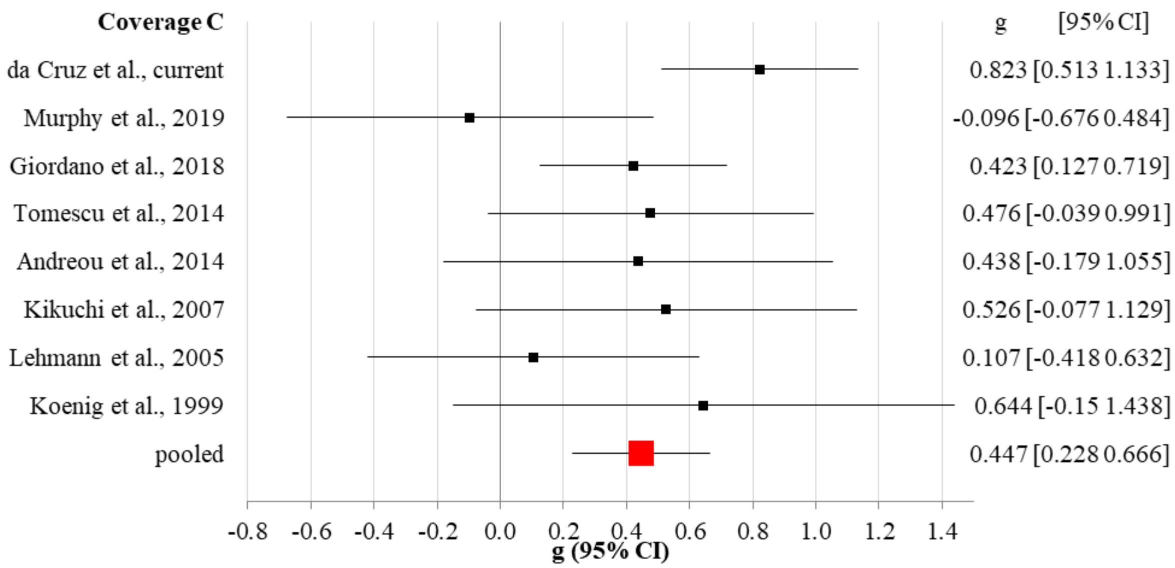

Supplementary Figure 8 - Forest plot of studies considering the time coverage of microstate class C. A two-sided *Z*-test revealed that patients have significantly longer microstate class C time coverage than controls (N=647, k=8, g=0.447, 95% CI [0.228, 0.666], p=6.304e-5, p_{holm}=6.934e-4). *P*-values were corrected for 12 comparisons (3 microstate parameters × 4 microstate classes) using Bonferroni-Holm correction (p_{holm}). I^2 statistics suggests that there is low variability among studies (i.e., heterogeneity; I^2=35%, p=0.148). Data are presented as Hedge's g and error bars indicate 95% CI. Source data are provided as a Source Data file.

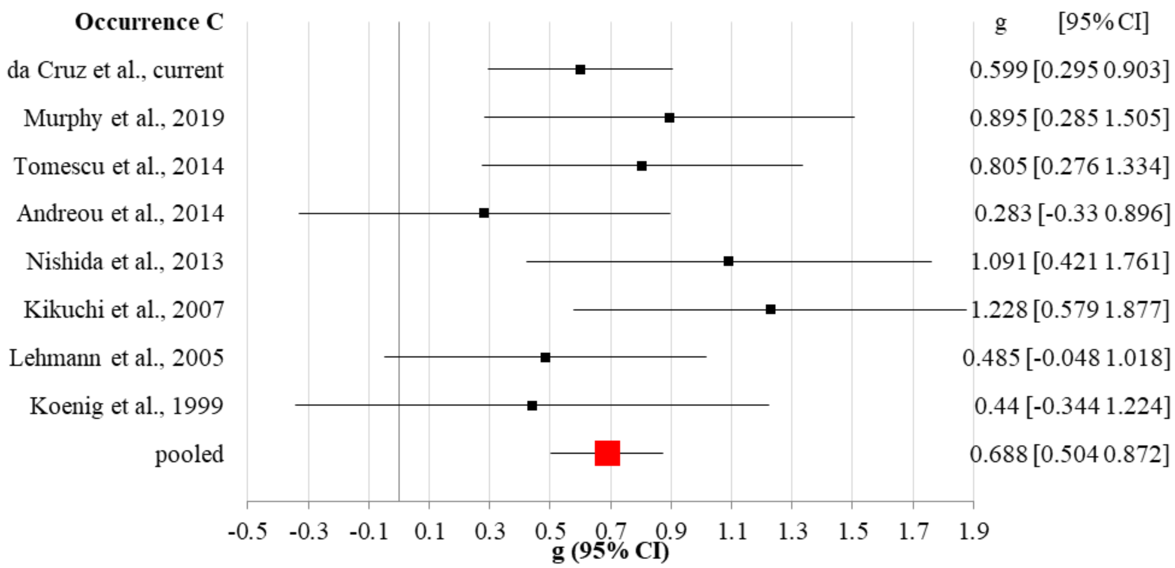

Supplementary Figure 9 - Forest plot of studies considering the occurrence of microstate class. A two-sided *Z*-test revealed that microstate class C occurs significantly more in patients than controls C (N=479, k=8, *g*=0.688, 95% CI [0.504, 0.872], *p*=2.430e-13, p_{holm}=2.916e-12). *P*-values were corrected for 12 comparisons (3 microstate parameters × 4 microstate classes) using Bonferroni-Holm correction (p_{holm}). I^2 statistics suggests that there is low variability among studies (i.e., heterogeneity; I^2=8%, *p*=0.367). Data are presented as Hedge’s g and error bars indicate 95% CI. Source data are provided as a Source Data file.

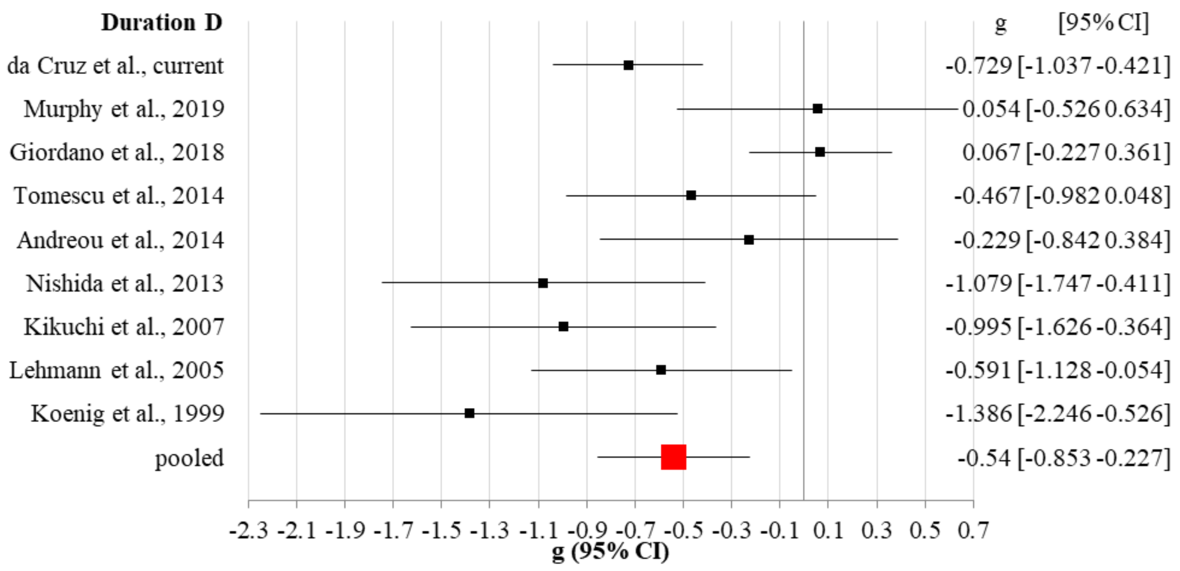

Supplementary Figure 10 - Forest plot of studies considering the mean duration of microstate class D. A two-sided *Z*-test revealed that patients have significantly shorter microstate class D mean durations than controls (N=685, k=9, g=-0.540, 95% CI [-0.853, -0.227], p=7.170e-4, p_{holm}=0.007). *P*-values were corrected for 12 comparisons (3 microstate parameters × 4 microstate classes) using Bonferroni-Holm correction (p_{holm}). I^2 statistics suggests that there is medium variability among studies (i.e., heterogeneity; I^2=73%, p=0.0003). Data are presented as Hedge's g and error bars indicate 95% CI. Source data are provided as a Source Data file.

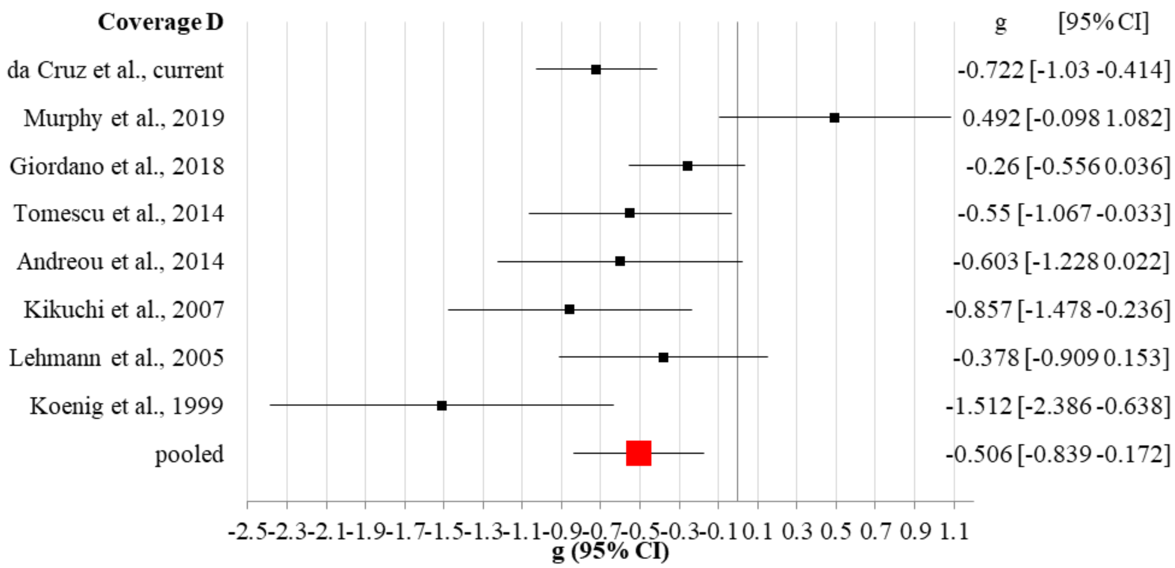

Supplementary Figure 11 - Forest plot of studies considering the time coverage of microstate class D. A two-sided *Z*-test revealed that patients have significantly shorter microstate class D time coverage than controls (N=647, k=8, g=-0.506, 95% CI [-0.839, -0.172], p=0.003, p_{holm}=0.027). *P*-values were corrected for 12 comparisons (3 microstate parameters × 4 microstate classes) using Bonferroni-Holm correction (p_{holm}). I^2 statistics suggests that there is medium variability among studies (i.e., heterogeneity; I^2=68%, p=0.002). Data are presented as Hedge's g and error bars indicate 95% CI. Source data are provided as a Source Data file.

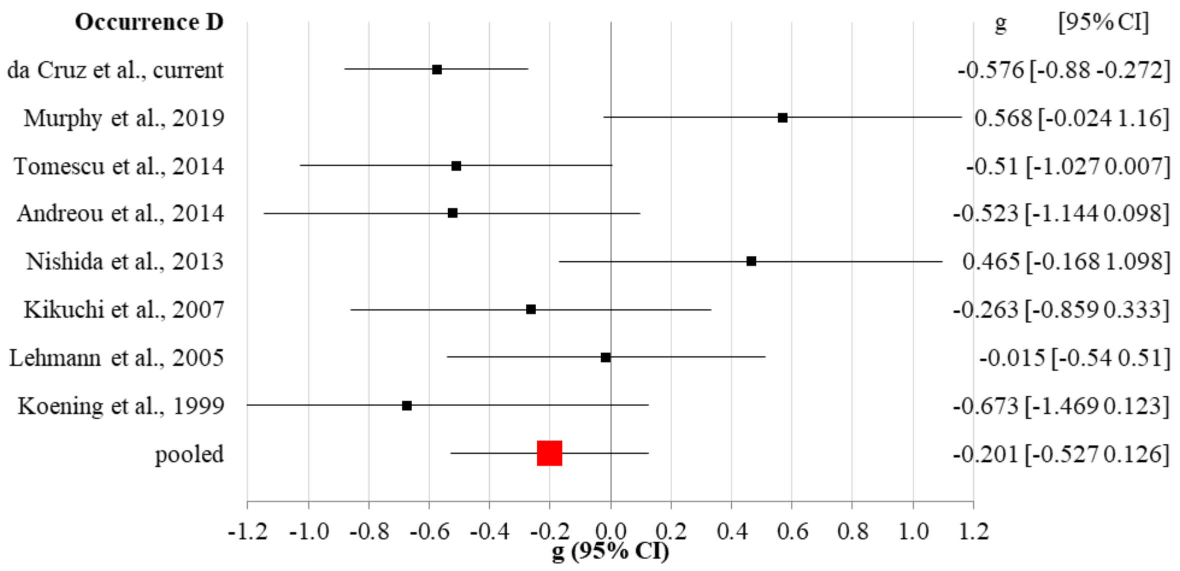

Supplementary Figure 12 - Forest plot of studies considering the occurrence of microstate class D. A two-sided *Z*-test revealed no consistent group differences between patients and controls (N=479, k=8, g=-0.201, 95% CI [-0.527, 0.126], p=0.228, p_{holm}=1.000). *P*-values were corrected for 12 comparisons (3 microstate parameters × 4 microstate classes) using Bonferroni-Holm correction (p_{holm}). I^2 statistics suggests that there is medium variability among studies (i.e., heterogeneity; I^2=65%, p=0.005). Data are presented as Hedge's g and error bars indicate 95% CI. Source data are provided as a Source Data file.

Supplementary References

1. Murray, M. M., Brunet, D. & Michel, C. M. Topographic ERP Analyses: A Step-by-Step Tutorial Review. *Brain Topogr.* **20**, 249–264 (2008).
2. Koenig, T., Stein, M., Grieder, M. & Kottlow, M. A Tutorial on Data-Driven Methods for Statistically Assessing ERP Topographies. *Brain Topogr.* **27**, 72–83 (2014).
3. Wagenmakers, E.-J. A practical solution to the pervasive problems of p values. *Psychon. Bull. Rev.* **14**, 779–804 (2007).
4. Rouder, J. N., Morey, R. D., Speckman, P. L. & Province, J. M. Default Bayes factors for ANOVA designs. *J. Math. Psychol.* **56**, 356–374 (2012).
5. Ly, A., Verhagen, J. & Wagenmakers, E.-J. Harold Jeffreys's default Bayes factor hypothesis tests: Explanation, extension, and application in psychology. *J. Math. Psychol.* **72**, 19–32 (2016).
