# Supplementary material for: EEG microstates are a candidate endophenotype for schizophrenia
Source: Nat Commun. 2020 Jun 18;11:3089. doi: 10.1038/s41467-020-16914-1 (PMC7303216; doi:10.1038/s41467-020-16914-1)
Supplement: Supplementary file 3 — Reporting Summary [file 41467_2020_16914_MOESM3_ESM.pdf]

nature research

Corresponding author(s): Janir Ramos da Cruz

Last updated by author(s): May 15, 2020

Reporting Summary

Nature Research wishes to improve the reproducibility of the work that we publish. This form provides structure for consistency and transparency in reporting. For further information on Nature Research policies, see Authors & Referees and the Editorial Policy Checklist.

Statistics

For all statistical analyses, confirm that the following items are present in the figure legend, table legend, main text, or Methods section.

n/a	Confirmed	
☐	☒	The exact sample size (*n*) for each experimental group/condition, given as a discrete number and unit of measurement
☐	☒	A statement on whether measurements were taken from distinct samples or whether the same sample was measured repeatedly
☐	☒	The statistical test(s) used AND whether they are one- or two-sided<br>*Only common tests should be described solely by name; describe more complex techniques in the Methods section.*
☐	☒	A description of all covariates tested
☐	☒	A description of any assumptions or corrections, such as tests of normality and adjustment for multiple comparisons
☐	☒	A full description of the statistical parameters including central tendency (e.g. means) or other basic estimates (e.g. regression coefficient) AND variation (e.g. standard deviation) or associated estimates of uncertainty (e.g. confidence intervals)
☐	☒	For null hypothesis testing, the test statistic (e.g. *F*, *t*, *r*) with confidence intervals, effect sizes, degrees of freedom and *P* value noted<br>*Give P values as exact values whenever suitable.*
☐	☒	For Bayesian analysis, information on the choice of priors and Markov chain Monte Carlo settings
☒	☐	For hierarchical and complex designs, identification of the appropriate level for tests and full reporting of outcomes
☐	☒	Estimates of effect sizes (e.g. Cohen's *d*, Pearson's *r*), indicating how they were calculated

Our web collection on statistics for biologists contains articles on many of the points above.

Software and code

Policy information about availability of computer code

Data collection	Data was collected with Biosemi ActiView 605.
Data analysis	Cartool 3.70, JASP (version 0.12.1), R (version 3.6.1), OpenMeta Analyst, APP

For manuscripts utilizing custom algorithms or software that are central to the research but not yet described in published literature, software must be made available to editors/reviewers. We strongly encourage code deposition in a community repository (e.g. GitHub). See the Nature Research guidelines for submitting code & software for further information.

Data

Policy information about availability of data

All manuscripts must include a data availability statement. This statement should provide the following information, where applicable:

- Accession codes, unique identifiers, or web links for publicly available datasets
- A list of figures that have associated raw data
- A description of any restrictions on data availability

The data that support the findings of this study are available upon reasonable request. The source data underlying Figure 1a-d, Figure 2a-d, and Figure 3a-c and Supplementary Figures 1, 2, 3, 4, 5, 6, 7, 8, 9, 10, 11, and 12 are provided as a Source Data File.

Field-specific reporting

Please select the one below that is the best fit for your research. If you are not sure, read the appropriate sections before making your selection.

☐ Life sciences ☒ Behavioural & social sciences ☐ Ecological, evolutionary & environmental sciences

October 2018

Behavioural & social sciences study design

All studies must disclose on these points even when the disclosure is negative.

Study description

Quantitative study with resting-state EEG data of schizophrenia patients, their siblings, patients with a first episodes of psychosis, and healthy controls.

Research sample

All participants have participated in a previous study on masking and evoked-related potentials (ERPs). Masking and ERP data of some participants have been already published, while data of other participants have not been analyzed yet. Resting microstate dynamics data of 27 patients with schizophrenia and 27 healthy controls have already been published in previous work (Tomescu et al., 2015). Masking and ERP data of 89 patients with schizophrenia, 39 siblings, and 63 controls have already been published (da Cruz et al., 2020; Favrod et al., 2019). Masking and ERP data of 21 of patients with a first episodes of psychosis (FEP) have been published in previous work (Favrod et al., 2018).

101 patients with schizophrenia (11 females; 95 right-handed; mean age: 36.9 ± 8.8; recruited from the Tbilisi Mental Health Hospital or the psycho-social rehabilitation center), 43 siblings of patients with schizophrenia (21 females; 41 right-handed; mean age: 31.8 ± 10.4; recruited from the Tbilisi Mental Health Hospital or the psycho-social rehabilitation center), and 75 healthy controls (39 females; 71 right-handed; mean age: 35.1 ± 7.7; residents of Tbilisi, Georgia) participated in Study 1.

22 FEP (12 females; 21 right-handed; mean age: 29.6 ± 9.1; recruited from the Tbilisi Mental Health Hospital or the Acute Psychiatric Departments of Multiprofile Clinics) and 22 (8 females; 21 right-handed; mean age: 31.3 ± 10.1; pseudo-randomly selected from our pool of 101 patients with schizophrenia patients, to match the 22 FEP as closely as possible, regarding gender, age, and education).

Patients were invited to participate in the study when they had recovered sufficiently from an acute psychotic episode. Patients were diagnosed using the Diagnostic and Statistical Manual of Mental Disorders Fourth Edition (DSM-IV) by means of an interview based on the SCID-CV (Structured Clinical Interview for DSM-IV, Clinician Version), information from staff, and study of patients' records. Psychopathology of patients with schizophrenia and FEP was assessed by an experienced psychiatrist using the Scales for the Assessment of Negative Symptoms (SANS) and Scales for the Assessment of Positive Symptoms (SAPS).

We included siblings of the patients with schizophrenia only when they had no history of psychoses. Controls were recruited from the general population, aiming to match patients and siblings as closely as possible. All siblings and controls were free from psychiatric axis I disorders. Family history of psychosis was an exclusion criterion for the control group. General exclusion criteria were alcohol or drug abuse, severe neurological incidents or diagnoses (including head injury), development disorders (autism spectrum disorder or intellectual disability) or other somatic mind-altering illnesses, assessed through interview by certified psychiatrists.

Since schizophrenia is a heterogeneous disease, our samples are small to be representative of the full schizophrenia spectrum and their siblings. Because controls were recruited to match patients and their patients demographically, the sample is not representative of the whole population.

References:

Tomescu et al. (2015). Schizophrenia patients and 22q11.2 deletion syndrome adolescents at risk express the same deviant patterns of resting state EEG microstates: A candidate endophenotype of schizophrenia. Schizophrenia Research: Cognition.

Favrod et al. (2018). Electrophysiological correlates of visual backward masking in patients with first episode psychosis. Psychiatry Research: Neuroimaging.

da Cruz et al. (2020). Neural compensation mechanisms of siblings of schizophrenia patients as revealed by high-density EEG. Schizophrenia Bulletin

Favrod et al. (2019). Electrophysiological correlates of visual backward masking in patients with major depressive disorder. Psychiatry Research: Neuroimaging.

Sampling strategy

The sample was a convenience sample.

No statistical test was done to determine the sample size a priori. However, we conducted sensitivity power analyses with the program GPower (Erdfelder et al., 1996) to determine what level of effect we could find with the proposed samples.

For patients (n=101) vs controls (n=75), sensitivity analysis indicated a sensitivity to detect an effect size (Cohen's d) of around 0.429, with a power of 80% in a two-tailed independent group t-test with an alpha = 0.05. This effect size is similar to the minimum significant (not corrected for multiple comparisons) effect size (for mean duration of microstate class B, Hedge's g = 0.435) reported by Rieger and colleagues in a meta-analysis to estimate the effect size of EEG microstate abnormalities in schizophrenia patients compared to controls (Rieger et al., 2015).

For siblings (n=43) vs controls (n=75), sensitivity analysis indicated a sensitivity to detect an effect size (Cohen's d) of around 0.540, with a power of 80% in a two-tailed independent group t-test with an alpha = 0.05. This effect size is similar to the minimum significant (not corrected for multiple comparisons) effect size (for time of coverage of microstate class C, Hedge's g = 0.569) reported by Rieger and colleagues in the above-mentioned meta-analysis (Rieger et al., 2015), for the microstate classes whose abnormalities have been suggested to be a candidate endophenotype for schizophrenia (Classes C and D).

For patients (n=101) vs siblings (n=43), sensitivity analysis indicated a sensitivity to detect an effect size (Cohen's d) of around 0.514, with

a power of 80% in a two-tailed independent group t-test with an alpha = 0.05. According to Cohen, this is a medium effect size (Cohen, 1988).

For patients with a first episodes of psychosis (n=22) vs. matched chronic patients (n=22), we performed a sensitivity analysis to determine the interaction and main effect of group effect sizes that we can detect with a power of 80%, given 22 participants in each of the 2 groups and 4 microstate classes. The analysis revealed that we could detect interaction effects and main effects of group with main effect sizes with eta-squared of 0.068 and 0.026, which are medium and small effect sizes according to Cohen (Cohen, 1988). However, a sensitivity analysis indicated a sensitivity to detect an effect size (Cohen's d) of around 0.865, with a power of 80% in a two-tailed independent group t-test with an alpha = 0.05. According to Cohen , this is a large effect size (Cohen, 1988).

In sum, the sample size of schizophrenia patients, siblings, and healthy controls, in the current manuscript, are large enough to detect the effects of interest and they are much larger than the samples usually reported in the EEG microstates in schizophrenia literature. For the first episode of psychosis study, the sample size are not large enough to detect small group difference effects but the samples are within the normal range of sample sizes in the EEG microstate literature. In addition, the sample sizes in the first episode of psychosis study were sufficient to provide enough evidence for the null hypothesis (no difference between groups) in a Bayesian framework of analysis.

Erdfelder et al. (1996). GPOWER: A general power analysis program. Behav. Res. Methods Instrum. Comput.

Rieger et al. (2016). 15 Years of Microstate Research in Schizophrenia – Where Are We? A Meta-Analysis. Front. Psychiatry.

Cohen (1988). Statistical Power Analysis for the Behavioral Sciences.

Data collection	Participants were sitting in a dim lit room. They were instructed to keep their eyes closed and to relax for five minutes. Resting-state EEG was recorded before participants participated in a masking experiment and using a BioSemi Active 2 system (Biosemi) with 64 Ag-AgCl sintered active electrodes, referenced to the common mode sense (CMS) electrode. Only the experimenter was with the participants during the experiment. The experimenter was aware of the hypothesis for the masking experiment but blind to the hypothesis for the resting-state EEG microstate study.
Timing	Start date: 2010-06-14<br>Stop date: 2019-11-28
Data exclusions	No participants were excluded from the analysis.
Non-participation	For the first episode of psychosis (FEP) study, all the 22 FEP participants were invited to participate in all three sessions but six of them dropped out after the first experiment and other five patients dropped out after the second experiments. The reasons for non-participation were that 2 patients could not be reached by the contacts they provided, 6 patients were not living in Tblisi and refused to come to Tblisi just for the experiments, and the remaining ones just refused to participate without giving a specific reason.
Randomization	For the first episode of psychosis (FEP) study, from our pool of 101 patients with schizophrenia, we pseudo-randomly selected 22 patients (Patients_22), to match the 22 FEP as closely as possible, regarding gender, age, and education.<br><br>For patients vs. controls, groups differed in terms of gender and education, gender was used as a factor while education was used as a covariate in subsequent analyses.<br><br>For patients vs. siblings, since groups differed in terms of gender and gender, gender was used as a factor while age was used as a covariate in subsequent analyses.

Reporting for specific materials, systems and methods

We require information from authors about some types of materials, experimental systems and methods used in many studies. Here, indicate whether each material, system or method listed is relevant to your study. If you are not sure if a list item applies to your research, read the appropriate section before selecting a response.

Materials & experimental systems

n/a	Involved in the study	
☒	☐	Antibodies
☒	☐	Eukaryotic cell lines
☒	☐	Palaeontology
☒	☐	Animals and other organisms
☐	☒	Human research participants
☒	☐	Clinical data

Methods

n/a	Involved in the study	
☒	☐	ChIP-seq
☒	☐	Flow cytometry
☒	☐	MRI-based neuroimaging

Human research participants

Policy information about studies involving human research participants

Population characteristics	"See above"

Recruitment	Schizophrenia patients, their siblings, and first episode patients were recruited from the Tbilisi Mental Health Hospital, the psycho-social rehabilitation center, or the Acute Psychiatric Departments of Multiprofile Clinics. Patients were invited to participated in the study when they had recovered sufficiently from an acute psychotic episode. Controls were recruited from the general population, aiming to match patients and siblings as closely as possible.<br><br>In the longitudinal study of first episodes patients, we face an attrition bias. Since only half of the first episodes patients completed the 3 testing session, we cannot draw firm conclusions on the stability of the microstate dynamics.
Ethics oversight	Ethical Committee of Institute of Postgraduate Medical Education and Continuous Professional Development (Georgia). Protocol number: 09/07. Title: "Genetic polymorphisms and early information processing in schizophrenia"

Note that full information on the approval of the study protocol must also be provided in the manuscript.
